# Congenital Anomaly–Related Mortality in Children Aged 0 to 14 Years in the US

**DOI:** 10.1001/jamanetworkopen.2025.33523

**Published:** 2025-09-25

**Authors:** Guodong Ding, Ziru Shang, Yan Chen, Angela Vinturache, Yongjun Zhang

**Affiliations:** 1 Department of Pediatrics, Xinhua Hospital, Shanghai Jiao Tong University School of Medicine, Shanghai, China; 2 Department of Obstetrics and Gynecology, University of Alberta, Edmonton, Alberta, Canada

## Abstract

This cross-sectional study examines the mortality trends and sociodemographic disparities in congenital malformations among US children aged 0 to 14 years from 1999 to 2023.

## Introduction

Congenital anomalies remain a leading cause of childhood mortality in the US, consistently ranking among the top 5 causes of death for children aged 0 to 14 years.^1^ However, most research has focused on infants or relied on outdated data, limiting our understanding of the broader epidemiology during childhood.^2,3^ This study examined mortality trends and sociodemographic disparities in congenital malformations among children aged 0 to 14 years from 1999 to 2023.

## Methods

We analyzed mortality data from the CDC WONDER database for deaths with an underlying cause coded as congenital anomalies (*ICD-10* Q00-Q99) from 1999 through 2023.^4^ This study used deidentified, publicly available data; thus, informed consent was not required and the study was exempt from institutional review board oversight at Xinhua Hospital, Shanghai Jiao Tong University School of Medicine. This cross-sectional study followed the STROBE reporting guideline.

Records missing race and ethnicity were excluded from subgroup analyses. Trends in mortality rates were assessed using joinpoint regression software version 5.3.0 (National Cancer Institute) to calculate average annual percentage changes (AAPC) and annual percentage changes (APC). Statistical significance was set at a 2-tailed *P* < .05. Data were analyzed from February 15 to April 5, 2025. Additional methodological details are available in eMethods in Supplement 1.

## Results

**Figure 1.**
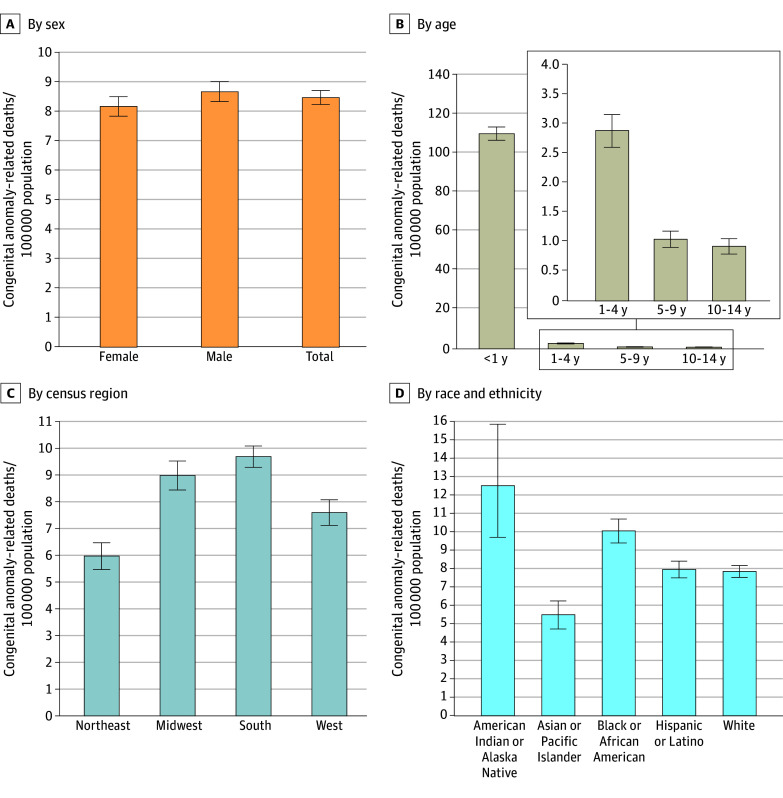
Comparison of Congenital Anomaly–Related Mortality Among Children Aged 0 to 14 Years, Stratified by Sex, Age, Census Region (2023), and Race and Ethnicity (2020) Comparisons of congenital anomaly–related mortality rates among children aged 0 to 14 years, stratified by sex (A), age (B), census region (C), and race and ethnicity (D). Differences in mortality rates across sex, age (<1 year as reference), census regions (South as reference), and race and ethnicity (White as reference) were assessed using the 2-sample *z* test. There was no missing data for sex, age, or census region. However, for race and ethnicity, some records for the Hispanic category were marked as not stated. As a result, a total of 20 deaths were excluded from the race and ethnicity stratified analysis in 2020. Data were obtained from the US Centers for Disease Control and Prevention Wide-Ranging Online Data for Epidemiologic Research. We limited our results stratified by race and ethnicity to 2020 due to changes in the classification of races after 2020. All mortality rates were presented per 100 000 population. Error bars represent the 95% CIs.

Between 1999 and 2023, 146 398 congenital anomaly–related deaths were recorded among children aged 0 to 14 years, with an overall mortality rate of 9.7 per 100 000. Across most years, males exhibited significantly higher mortality rates than females (eg, 8.69 vs 8.19 in 2023). Infants younger than 1 year consistently had the highest mortality rate (eg, 109.77 in 2023) compared with children aged 1 to 4 years (2.87), 5 to 9 years (1.04), and 10 to 14 years (0.92) in 2023 (Figure 1).

Across all years, the South (eg, 9.68) and Midwest (8.98) exhibited higher mortality rates, followed by the West (7.59) and Northeast (5.97) in 2023. American Indian or Alaska Native children (eg, 12.48) and Black or African American children (10.03) had higher mortality rates than Hispanic or Latino children (7.94), White children (7.83), and Asian or Pacific Islander children (5.48) in 2020. All differences were significant (*P* < .01).

**Figure 2.**
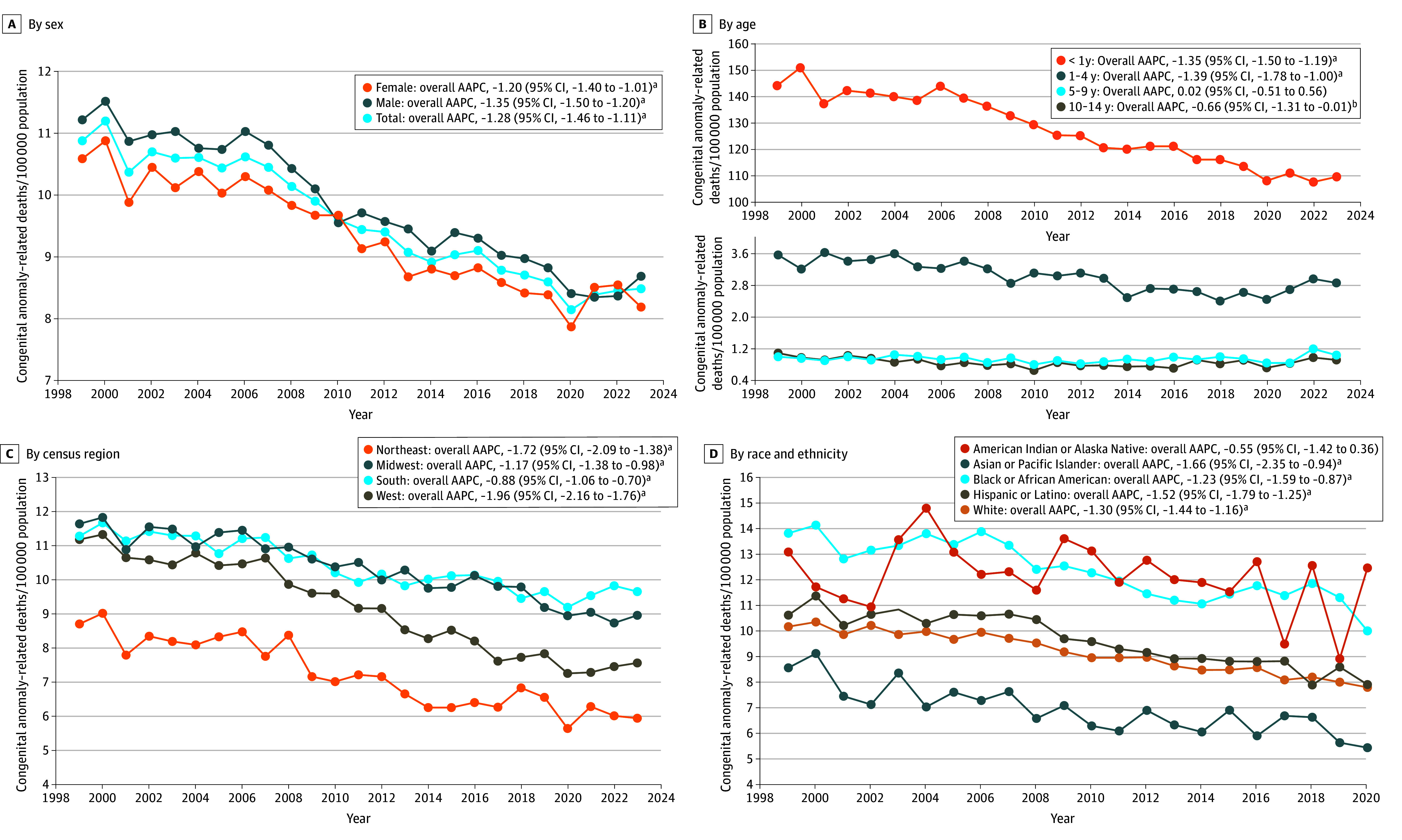
Trends in Congenital Anomaly–Related Mortality Among Children Aged 0 to 14 Years, Stratified by Sex, Age, Census Region (1999-2023), and Race and Ethnicity (1999-2020) No missing data for sex, age, or census region. Some records for the Hispanic category were marked as not stated; 827 deaths were excluded from the race and ethnicity stratified analysis. Race and ethnicity results were limited to 2020 due to changes in race classification after 2020. ^a^*P* < .001. ^b^*P* < .05.

The overall mortality rate declined from 10.89 in 1999 to 8.49 per 100 000 in 2023, corresponding to an AAPC of −1.28% (95% CI, −1.46% to −1.11%; *P* < .001) (Figure 2). A notable decrease occurred between 2007 (10.46) and 2013 (9.08), with an APC of −2.19% (95% CI, −3.45% to −0.92%; *P* = .002). Subgroup analyses showed similar downward trends, except for children aged 5 to 9 years and American Indian or Alaska Native children, where no significant trends were found.

## Discussion

This study revealed 2 principal findings. First, congenital anomaly–related mortality among children declined from 1999 to 2023. This decline reflects multifactorial advances in screening, diagnosis, interventions, and access to care. Second, current mortality rates were higher among boys, infants, American Indian or Alaska Native children, and residents of the South. The elevated mortality in individuals of Indigenous ancestry, consistent with prior reports,^5^ suggests structural barriers to care and warrants targeted investigation and intervention. Although the overall prevalence of congenital anomalies has remained stable,^6^ improved survival rates mean that more children with complex health needs are living into adulthood. This shift emphasizes the necessity for longitudinal care strategies and health equity efforts to support this growing population. Limitations include potential misclassification of cause of death and lack of stratification by congenital anomaly type. Continued efforts to improve the quality and accessibility of prenatal and pediatric care, coupled with targeted public health strategies, are crucial for reducing these disparities and supporting children with congenital anomalies throughout childhood and beyond.

## Notes

Supplement 1.
eMethods.

Supplement 2.
Data Sharing Statement
