# Supplementary material for: Congenital Anomaly–Related Mortality in Children Aged 0 to 14 Years in the US
Source: JAMA Netw Open. 2025 Sep 25;8(9):e2533523. doi: 10.1001/jamanetworkopen.2025.33523 (PMC12464788; doi:10.1001/jamanetworkopen.2025.33523)
Supplement: Supplement 1. — eMethods. [file jamanetwopen-e2533523-s001.pdf]

Supplemental Online Content

Ding G, Shang Z, Chen Y, Vinturache A, Zhang Y. Congenital anomaly-related mortality in children aged 0 to 14 years in the US. *JAMA Netw Open*. 2025;8(9):e2533523. doi:10.1001/jamanetworkopen.2025.33523

eMethods.

This supplemental material has been provided by the authors to give readers additional information about their work.

eMethods

The US Centers for Disease Control and Prevention (CDC) Wide-ranging Online Data for Epidemiologic Research (WONDER) is an online database that provides mortality statistics and population counts by state and cause of death, based on death certificates (1). The classification of congenital anomalies in this study is based on the *International Classification of Diseases*, 10th Revision (ICD-10), codes Q00-Q99 (2). The study aggregated all diagnoses within this classification and did not disaggregate by specific disorders. Using the CDC WONDER online portal, ICD-10 codes corresponding to congenital anomalies were entered under the "Underlying Cause of Death" query field to obtain mortality data from 1999 to 2023.

The population estimates were obtained annually from the US Census Bureau, which used bridged-race categories to ensure consistency across different census years and incorporated census counts along with adjustments for race reporting differences. The population estimates for 1999 were derived from an intercensal series based on the 1990 and 2000 censuses. Estimates for 2000 and 2010 were based on the respective US Census counts for those years. The estimates for 2001–2009 were based on an intercensal series incorporating the 2000 and 2010 Census counts. Population estimates for 2011–2020 were derived from a postcensal series based on the 2010 Census. Estimates for 2021–2023 were based on a postcensal series using the 2020 Census. For our study, the population of interest is children aged 0–14 years during the given time period. We summed the population estimates for each year. For example, for the age group "0–14 years" from 1999 to 2000, we combined the population estimate for 1999 with that for 2000 to form the total population denominator for that time period. These combined population estimates are used to calculate the death rates. The study reported mortality rates per 100,000 population estimates, both overall and stratified by sex, age, race and ethnicity, and geographic region, with corresponding 95% confidence intervals (CIs). We limited our results stratified by race and ethnicity to 2020 due to changes in the classification of races after 2020.

Annual population denominators for children aged 0 to 14 years were derived from US Census Bureau estimates, aggregated yearly to calculate mortality rates per 100 000 population, along with 95% CIs. We stratified deaths by age at the time of death (younger than 1 year, 1 to 4

© 2025 Ding G et al. *JAMA Network Open.*

years, 5 to 9 years, and 10 to 14 years), sex, race (American Indian or Alaska Native, Asian or Pacific Islander, Black, and White) and ethnicity (Hispanic, non-Hispanic), and US Census region.

Age was categorized into four groups: under 1 year, 1-4 years, 5-9 years, and 10-14 years. Race and ethnicity were categorized into four non-Hispanic racial groups—American Indian or Alaska Native, Asian or Pacific Islander, Black or African American, and White—along with the Hispanic ethnicity category (of any race). Geographic regions were defined according to the US Census Bureau's classification, which includes the Northeast, Midwest, South, and West regions (3).

In our study, we used the Joinpoint regression software version 5.3.0 (National Cancer Institute) to calculate the average annual percent change (AAPC) with 95% CIs to assess the average annual change in mortality rates for congenital malformations from 1999 to 2023. This calculation was done both overall and for subgroups stratified by sex, age group, census regions, and race and ethnicity. The AAPC summarizes the overall average change in mortality rates over the entire study period, providing a single value that represents the annual rate of change. The annual percent change (APC) refers to the annual percent change, which represents the change in the mortality rate from one year to the next. Joinpoint regression was used to calculate these annual changes, identify years with significant shifts in the trend, and determine the slopes for each time segment. This approach helps pinpoint when significant changes in trends occurred over the study period. The program started with 0 joinpoints and tested whether adding 1 or more joinpoints (up to 4 in our study) would significantly improve the model. For this study, we had between 22 and 26 data points, and the default maximum number of joinpoints was set to 4.

The US CDC's WONDER database offers several strengths, such as extensive national coverage, providing valuable public health data on mortality, morbidity, and demographics, which enables trend analysis over time. Its user-friendly interface makes it accessible to researchers, and the dataset is nationally representative, ensuring broad applicability. However, limitations include the potential misclassification of causes of death, a lack of detailed individual-level data, and sometimes insufficient geographical granularity. Additionally, data completeness may vary, and there could be delays in the release of the most recent data, which may hinder real-time analysis.

© 2025 Ding G et al. *JAMA Network Open*.

eTable: ICD-10 Codes Used to Define Congenital Anomaly-Related Deaths

ICD-10 Code	Description
**Q00-Q07**	**Congenital malformations of the nervous system**
Q00	Anencephaly and similar malformations
Q01	Encephalocele
Q02	Microcephaly
Q03	Congenital hydrocephalus
Q04	Other congenital malformations of brain
Q05	Spina bifida
Q06	Other congenital malformations of spinal cord
Q07	Other congenital malformations of nervous system
**Q10-Q18**	**Congenital malformations of eye, ear, face and neck**
Q10	Congenital malformations of eyelid, lacrimal apparatus and orbit
Q11	Anophthalmos, microphthalmos and macrophthalmos
Q12	Congenital lens malformations
Q13	Congenital malformations of anterior segment of eye
Q14	Congenital malformations of posterior segment of eye
Q15	Other congenital malformations of eye
Q16	Congenital malformations of ear causing impairment of hearing
Q17	Other congenital malformations of ear
Q18	Other congenital malformations of face and neck
**Q20-Q28**	**Congenital malformations of the circulatory system**
Q20	Congenital malformations of cardiac chambers and connections
Q21	Congenital malformations of cardiac septa
Q22	Congenital malformations of pulmonary and tricuspid valves
Q23	Congenital malformations of aortic and mitral valves
Q24	Other congenital malformations of heart
Q25	Congenital malformations of great arteries
Q26	Congenital malformations of great veins
Q27	Other congenital malformations of peripheral vascular system
Q28	Other congenital malformations of circulatory system
**Q30-Q34**	**Congenital malformations of the respiratory system**
Q30	Congenital malformations of nose
Q31	Congenital malformations of larynx
Q32	Congenital malformations of trachea and bronchus
Q33	Congenital malformations of lung
Q34	Other congenital malformations of respiratory system
**Q35-Q37**	**Cleft lip and cleft palate**
Q35	Cleft palate
Q36	Cleft lip
Q37	Cleft palate with cleft lip
**Q38-Q45**	**Other congenital malformations of the digestive system**
Q38	Other congenital malformations of tongue, mouth and pharynx
Q39	Congenital malformations of oesophagus
Q40	Other congenital malformations of upper alimentary tract
Q41	Congenital absence, atresia and stenosis of small intestine
Q42	Congenital absence, atresia and stenosis of large intestine

© 2025 Ding G et al. *JAMA Network Open*.

Q43	Other congenital malformations of intestine
Q44	Congenital malformations of gallbladder, bile ducts and liver
Q45	Other congenital malformations of digestive system
**Q50-Q56**	**Congenital malformations of genital organs**
Q50	Congenital malformations of ovaries, fallopian tubes and broad ligaments
Q51	Congenital malformations of uterus and cervix
Q52	Other congenital malformations of female genital organs
Q53	Undescended testicle
Q54	Hypospadias
Q55	Other congenital malformations of male genital organs
Q56	Indeterminate sex and pseudohermaphroditism
**Q60-Q64**	**Congenital malformations of the urinary system**
Q60	Renal agenesis and other reduction defects of kidney
Q61	Cystic kidney disease
Q62	Congenital obstructive defects of renal pelvis and congenital malformations of ureter
Q63	Other congenital malformations of kidney
Q64	Other congenital malformations of urinary system
**Q65-Q79**	**Congenital malformations and deformations of the musculoskeletal system**
Q65	Congenital deformities of hip
Q66	Congenital deformities of feet
Q67	Congenital musculoskeletal deformities of head, face, spine and chest
Q68	Other congenital musculoskeletal deformities
Q69	Polydactyly
Q70	Syndactyly
Q71	Reduction defects of upper limb
Q72	Reduction defects of lower limb
Q73	Reduction defects of unspecified limb(s)
Q74	Other congenital malformations of limb(s)
Q75	Other congenital malformations of skull and face bones
Q76	Congenital malformations of spine and bony thorax
Q77	Osteochondrodysplasia with defects of growth of tubular bones and spine
Q78	Other osteochondrodysplasias
Q79	Congenital malformations of musculoskeletal system, not elsewhere classified
**Q80-Q89**	**Other congenital malformations**
Q80	Congenital ichthyosis
Q81	Epidermolysis bullosa
Q82	Other congenital malformations of skin
Q83	Congenital malformations of breast
Q84	Other congenital malformations of integument
Q85	Phakomatoses, not elsewhere classified
Q86	Congenital malformation syndromes due to known exogenous causes, not elsewhere classified
Q87	Other specified congenital malformation syndromes affecting multiple systems
Q89	Other congenital malformations, not elsewhere classified
**Q90-Q99**	**Chromosomal abnormalities, not elsewhere classified**
Q90	Down syndrome
Q91	Edwards syndrome and Patau syndrome
Q92	Other trisomies and partial trisomies of the autosomes, not elsewhere classified
Q93	Monosomies and deletions from the autosomes, not elsewhere classified
Q95	Balanced rearrangements and structural markers, not elsewhere classified

© 2025 Ding G et al. *JAMA Network Open*.

Q96	Turner syndrome
Q97	Other sex chromosome abnormalities, female phenotype, not elsewhere classified
Q98	Other sex chromosome abnormalities, male phenotype, not elsewhere classified
Q99	Other chromosome abnormalities, not elsewhere classified

References

1. CDC WONDER. National Center for Health Statistics. About Underlying Cause of Death, 1999-2020. April 08, 2025. Accessed April 10, 2025. Available at: https://wonder.cdc.gov/ucd-icd10.html.

2. Okobi OE, Ibanga IU, Egbujo UC, Egbuchua TO, Oranu KP, Oranika US. Trends and Factors Associated With Mortality Rates of Leading Causes of Infant Death: A CDC Wide-Ranging Online Data for Epidemiologic Research (CDC WONDER) Database Analysis. Cureus. 2023; 15(9): e45652. doi: 10.7759/cureus.45652.

3. Ingram DD, Franco SJ. 2013 NCHS urban–rural classification scheme for counties. National Center for Health Statistics. Vital Health Stat 2(166). April 2014. Accessed April 10, 2025. Available at: https://www.cdc.gov/nchs/data/series/sr_02/sr02_166.pdf.

© 2025 Ding G et al. *JAMA Network Open.*
