# Supplementary material for: Congenital Anomaly–Related Mortality in Children Aged 0 to 14 Years in the US
Source: JAMA Netw Open. 2025 Sep 25;8(9):e2533523. doi: 10.1001/jamanetworkopen.2025.33523 (PMC12464788; doi:10.1001/jamanetworkopen.2025.33523)
Supplement: Supplement 2. — Data Sharing Statement [file jamanetwopen-e2533523-s002.pdf]

Data Sharing Statement

Ding. Congenital Anomaly-Related Mortality in Children Aged 0 to 14 Years in the US. *JAMA Netw Open*. Published September 23, 2025. doi:10.1001/jamanetworkopen.2025.33523

Data
Data available: Yes
Data types: Deidentified participant data
How to access data: Data sharing will be available from YZ upon a reasonable request. Electronic address: zhangyongjun@sjtu.edu.cn.
When available: beginning date: 12-01-2025, end date: 12-01-2026

Supporting Documents
Document types: Statistical/analytic code
How to access documents: Data sharing will be available from YZ upon a reasonable request. Electronic address: zhangyongjun@sjtu.edu.cn.
When available: beginning date: 12-01-2025, end date: 12-01-2026

Additional Information
Who can access the data: Data sharing will be available from YZ upon a reasonable request. Electronic address: zhangyongjun@sjtu.edu.cn.
Types of analyses: Data sharing will be available from YZ upon a reasonable request. Electronic address: zhangyongjun@sjtu.edu.cn.
Mechanisms of data availability: Data sharing will be available from YZ upon a reasonable request. Electronic address: zhangyongjun@sjtu.edu.cn.
Any additional restrictions: Data sharing will be available from YZ upon a reasonable request. Electronic address: zhangyongjun@sjtu.edu.cn.
